# Index of Alpha/Theta Ratio of the Electroencephalogram: A New Marker for Alzheimer’s Disease

**DOI:** 10.3389/fnagi.2013.00060

**Published:** 2013-10-09

**Authors:** Magali T. Schmidt, Paulo A. M. Kanda, Luis F. H. Basile, Helder Frederico da Silva Lopes, Regina Baratho, Jose L. C. Demario, Mario S. Jorge, Antonio E. Nardi, Sergio Machado, Jéssica N. Ianof, Ricardo Nitrini, Renato Anghinah

**Affiliations:** ^1^Division of Clinical Neurology, Referral Center for Cognitive Disorders, HCFMUSP, São Paulo, Brazil; ^2^Laboratory of Psychophysiology, Faculty of Health, Universidade Metodista de São Paulo, São Paulo, Brazil; ^3^Division of Neurosurgery, Department of Neurology, University of São Paulo Medical School, São Paulo, Brazil; ^4^Economy School of Pontifical Catholic University of São Paulo, São Paulo, Brazil; ^5^Panic and Respiration Laboratory, Institute of Psychiatry, Federal University of Rio de Janeiro (IPUB/UFRJ), Rio de Janeiro, Brazil; ^6^National Institute of Translational Medicine (INCT-TM), Rio de Janeiro, Brazil; ^7^Division of Neurology, Department of Neurology, University of São Paulo Medical School, São Paulo, Brazil

**Keywords:** alpha, theta, Alzheimer’s disease, EEG, logistic regression

## Abstract

**Objective:** We evaluated quantitative EEG measures to determine a screening index to discriminate Alzheimer’s disease (AD) patients from normal individuals.

**Methods:** Two groups of individuals older than 50 years, comprising a control group of 57 normal volunteers and a study group of 50 patients with probable AD, were compared. EEG recordings were obtained from subjects in a wake state with eyes closed at rest for 30 min. Logistic regression analysis was conducted.

**Results:** Spectral potentials of the alpha and theta bands were computed for all electrodes and the alpha/theta ratio calculated. Logistic regression of alpha/theta of the mean potential of the C3 and O1 electrodes was carried out. A formula was calculated to aid the diagnosis of AD yielding 76.4% sensitivity and 84.6% specificity for AD with an area under the ROC curve of 0.92.

**Conclusion:** Logistic regression of alpha/theta of the spectrum of the mean potential of EEG represents a good marker discriminating AD patients from normal controls.

## Introduction

Alzheimer’s disease (AD) is the most common form of dementia, comprising 50–70% of all dementia cases. The burden of dementia on the public health is increasing rapidly as our population ages (Kukull and Bowen, 2002).

At present, no single neuropsychological test or complementary exam is able to provide a reliable diagnosis of the disease in its early stages (Nitrini et al., 2005). The inclusion of clinical neurophysiological techniques, or more specifically, electroencephalography, in diagnostic research protocols for AD is wholly justified given EEG’s wide availability, low cost, and high sensitivity, allowing serial exams and neurological evolution follow-up to be performed.

In addition to visual and spectral analyses, quantitative EEG offers other research tools such as the alpha/theta ratio, an index which shows the percentage of alpha versus theta spectral potential during wake state at rest. The normative value of the ratio in healthy individuals is defined as ≥1. The study by Cibils (2002) confirmed a lower alpha/theta ratio in patients with early and moderate stages of AD, suggesting a pattern of activity for AD of increased theta and decreased alpha.

However, the detection rate (sensitivity) of AD by qEEG lays in the 60–90% range (among groups), depending on disease stage and method used (Anderer et al., 1994; Besthorn et al., 1997). The search for EEG-based methods able to discriminate normal individuals from demented patients remains a challenge. All of the studies mentioned above provided the basis for the present study (Anderer et al., 1994; Besthorn et al., 1997). Thus, our aim was to determine a screening index to discriminate AD patients from normal individuals for use in routine clinical practice to aid the diagnosis of AD.

## Materials and Methods

### Subjects

The sample comprised 107 subjects (48 men and 59 women) included in a comparative assessment involving two groups (experimental group and control group). The experimental group comprised 50 patients (19 men and 31 women) diagnosed with probable AD, according to NINCDS-ADRDA criteria (McKhann et al., 1984), of mild to moderate severity, based on DSM-IV R criteria, and with scores on the MMSE of between 12 and 25 points (Ordinance of The Health Care Secretariat/Brazilian Ministry of Health, 2002). All patients were recruited from the Outpatient Clinic of the Cognitive and Behavioral Neurology Group (GNCC) of the Division of Clinical Neurology of the HCFMUSP and/or from the CEREDIC of the HCFMUSP, and were submitted to routine outpatient assessment for diagnosing AD (Caramelli et al., 2011). The control group comprised normal subjects (29 men and 28 women) with scores on the MMSE ≥26 and older than 50 years, drawn from a database held by the neurophysiology service of the Referral Center for Cognitive Disorders (CEREDIC) (Caramelli et al., 1999).

Subjects with a history of diabetes mellitus, nephropathies, thyroidopathies, alcoholism, hepatopathies, pulmonary diseases, and lack of vitamin B12 were excluded from the study.

### Standard protocol approvals, registrations, and subjects consent

The Research Ethics Committee of the Faculty of Medicine of University of São Paulo (FMUSP) approved all procedures (protocol number – 0895:09) and written informed consent was obtained from all the subjects enrolled on the study (signed by the participant or the guardian).

### EEG recording

Recordings during relaxed wakefulness were obtained from subjects seated in a comfortable chair – to minimize muscular artifacts – with eyes closed during 30 min. Data acquisition was carried out in an unlit room to reduce sensory interference. Briefly, 22 Ag/AgCl EEG electrodes were placed on the scalp placed in accordance with norms of the Brazilian Society of Clinical Neurophysiology (International 10–20 system with bi-auricular referential electrodes) for acquisition of the EEG (Anghinah et al., 2000). Electrode impedances were closely monitored and kept below 3 kΩ. Data were acquired using the Neurosoft system with 32 channels, 12-bit processor at a sampling frequency of ≥200 samples/channel/s with filters for high frequencies of 70 Hz (low pass) and low frequencies of 0.5 Hz (high pass).

An EEG technician and neurophysiologist were present during the entire recording session to observe the behavioral state of the patient and to monitor on-line for signal quality.

### EEG data processing and analysis

The data from baseline EEG were processed off-line using the software of Neurosoft.

Possible sources of artifacts, such as blinking, muscle, and saccade-related artifacts, were first identified by visual inspection and subsequently by independent component analysis (ICA). Portions of the EEG recording influenced by blinking-related artifacts were removed by visual inspection as were components exhibiting similar contamination on ICA.

A 40-s trace free of artifacts was selected (several 10- to 15-s periods edited to produce a single 40-s file). The midline (Fz, Cz, Pz, and Oz), auricular (isoelectric referential points), and Fp1; Fp2 (due to eyebrow movement contamination) electrodes were all excluded from the statistical analysis.

Spectral potentials of the alpha and theta bands were computed separately for all electrodes and electroencephalograms. Subsequently, the alpha/theta ratio for these spectral potentials was calculated. Thus, a total of 20 electrodes common to the whole sample were analyzed. Correlation Analysis and graphical analysis were carried out on the variables Maximum, Mean, and Total Potential as well as Pathology. Logistic Regression was performed for all the variables and the ROC curve was obtained.

## Results

### Demographic characteristics

There were a total of 50 patients with probable AD and 57 normal subjects. In the experimental group, age ranged from 52 to 89 years. These subjects had a mean age of 75.2 (±8.08) years old. The level of education of this group ranged from 0 to 13 years and the score of MMSE ranged from 12 to 25. The patients were diagnosed with regards to the severity of dementia by using the Clinical Dementia Rating (CDR) scale (Morris, 1993). In the Alzheimer’s group, 34 patients had a CDR 1 (mild dementia) and 16 patients had a CDR 2 (moderate dementia). In the control group, age ranged from 57 to 86 years. These subjects had a mean age of 67.21 (±8.56) years old. The level of education of this group ranged from 2 to 20 years and the score of MMSE ranged from 26 to 30.

### Analyses of EEG variables

Among the variables studied, those that had greatest correlation with Pathology (healthy group versus AD group) showed a negative association with 1% significance and only 1% probability of error for: (a) alpha/theta ratio of maximum potential; (b) alpha/theta ratio of mean potential; and (c) alpha/theta ratio of total potential.

Detailed analysis revealed that individuals classified as AD patients based on the alpha/theta ratio of Maximum, Mean, and Total Potentials had lower values as a result of progressive slowing of EEG waves. Conversely, among normal individuals, these variables yielded higher values. Analysis of the variables with only weak correlation showed a significant positive correlation with Mean Frequency, albeit for few electrodes. For Asymmetry, no significant correlation was detected for any of the electrodes.

**Figure 1:**
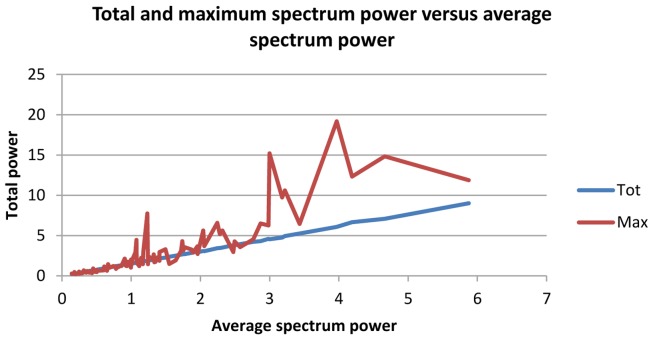
**Red line: mean of total potential of alpha/theta ratio of spectrum; yellow line: mean of maximum potential of alpha/theta ratio of spectrum**.

On overall statistical assessment, analysis of the correlation matrix showed highest values between Pathology and alpha/theta ratios for Maximum, Mean, and Total Potential, whereas alpha/theta ratio of Maximum Potential exhibited high variability, representing a disadvantage for use in patient classification. Therefore, the optimal variable for discriminating normal subjects from Alzheimer patients was Mean Potential, as depicted in the graph below and on the linear distribution (Figure 1).

Logistic regression analysis results indicated which variables best explained Pathology and yielded coefficient values for use in a future index to aid diagnosis of AD patients. Logistic regression was performed using both one variable and two variables to explain Pathology.

### Index 1

**Figure 2:**
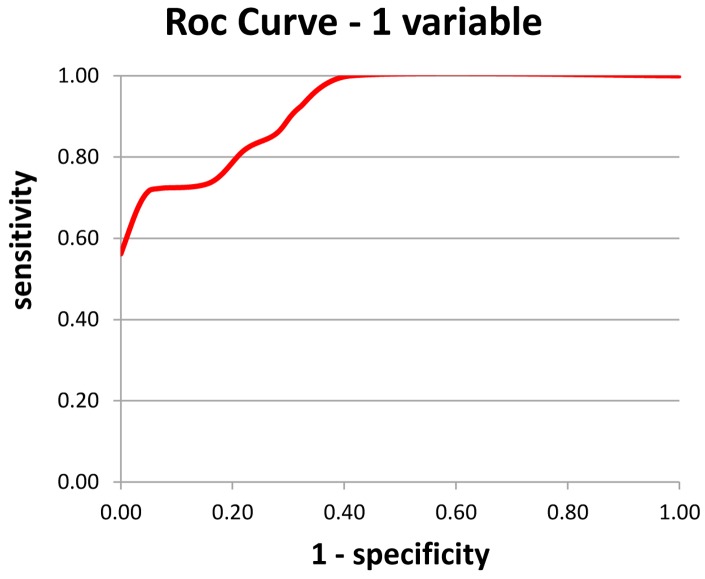
**Index 1 – using a single variable – the alpha/theta ratio of the mean potential of the O1 electrode; a: sensitivity and b: specificity**.

On the model with one variable, the alpha/theta ratio of Mean Potential for the O1 electrode (left) was chosen, explaining 77.6% of Pathology. Based on these results, a simple and fast Index (Index 1 – Figure 2) was calculated for aiding AD diagnosis.

An alpha/theta ratio of mean potential of O1 electrode >1.42 is deemed negative for AD; *b*-an alpha/theta ratio of mean potential of O1 electrode ≤1.42 is deemed positive for AD.

Use of Index 1 yielded 83 correct diagnoses out of 107 cases, or an overall rate of correct diagnosis of 77.6%.

Regarding sensitivity, among subjects classified as AD on the test, the result was correct in 73% of cases. In terms of specificity among subjects classified as Normal on the test, the result was correct in 82% of cases.

### Index 2

**Figure 3:**
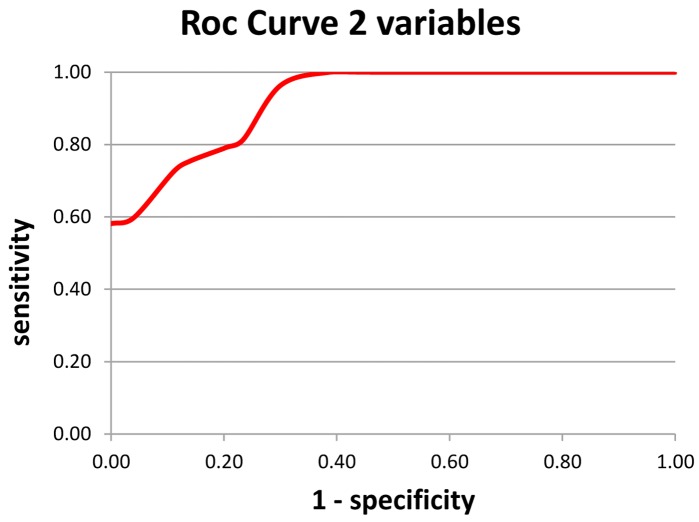
**Index 2 – using a double variable – the alpha/theta ratio of the mean potential of the O1 and C3 electrodes; a: sensitivity and b: specificity**.

Based on previous result, another formula was calculated for aiding AD diagnosis (Index 2 – Figure 3). The two variables chosen were alpha/theta ratio of Mean Potential of C3 electrode and alpha/theta ratio of Mean Potential of O1 electrode.

The Logistic regression analysis for the two variables model returns three coefficients “*a*,” ”*b*,” and “*c*.”

The model can be used to calculate the probability of the subject belonging to the normal group (*x* = 0) or the pathological group (*x* = 1). To compute the probability of belonging to the pathological group we must evaluate the following equation:

(1) $$P\left(x=1\right)=\frac{e^{\left(ax_{1}+bx_{2}+c\right)}}{1+e^{\left(ax_{1}+bx_{2}+c\right)}}$$

where:

*P*(*x* = 1) is the probability of belonging to the pathological group
*a* is the O1 electrode coefficient (*a* = −1.03844)
*x*_1_ is the alpha/theta ratio measured on the O1 electrode for the studied patient
*b* is the C3 electrode coefficient (*b* = −1.55758)
*x*_2_ is the alpha/theta ratio measured on the C3 electrode for the studied patient
*c* is the cut-off value *c* = 2.844023)
*e* is the natural logarithm base (*e* = 2.71828.)

The probability of belonging to the normal group can be computed as:

$$P\left(x=0\right)=1-P\left(x=1\right)$$

If *P*(*x* = 1) is >0.5 then *P*(*x* = 0) will be <0.5.

So we can write:

If *P*(*x* = 1) > 0.5 implies that the probability of belonging to pathological group is greater than the probability of belonging to the normal group.

But from Eq. 1 we can write:

If

$$\frac{e^{\left(ax_{1}+bx_{2}+c\right)}}{1+e^{\left(ax_{1}+bx_{2}+c\right)}}>0.5$$

implies that the probability of belonging to pathological group is greater than the probability of belonging to the normal group.

Multiplying the both sides of the inequality by:

$$1+e^{\left(ax_{1}+bx_{2}+c\right)}$$

Results:

$$e^{\left(ax_{1}+bx_{2}+c\right)}>0.5\left(1+e^{\left(ax_{1}+bx_{2}+c\right)}\right)$$

Multiplying the both sides of the inequality by two results:

$$2e^{\left(ax_{1}+bx_{2}+c\right)}>1+e^{\left(ax_{1}+bx_{2}+c\right)}$$

Then, If

$$e^{\left(ax_{1}+bx_{2}+c\right)}>1$$

implies that the probability of belonging to pathological group is greater than the probability of belonging to the normal group.

Applying the natural logarithm to the both sides of the inequality:

$$ax_{1}+bx_{2}+c>0$$

Replacing the coefficients:

$$\begin{array}{ccc} a & =-1.03844 & \\ b & =-1.55758\\ c & =2.844023 \end{array}$$

We can rewrite:

$$-1.03844x_{1}-1.55758x_{2}+2.844023>0$$

If

$$1.03844x_{1}+1.55758x_{2}<2.844023$$

implies that the probability of belonging to pathological group is greater than the probability of belonging to the normal group.

However these coefficients are difficult to remember.

If we multiply the both sides by a constant *k* > 0 the inequality remains unaltered.

Particularly if *k* = 11.6 we have:

$$12.0459x_{1}+18.0679x_{2}<32.99066$$

But 12.0459 is very close to 12
and 18.0679 is very close to 18
and 32.99066 is very close 33

These numbers are easy to remember and the expression is equivalent to the original equation. In fact, if:

$$12x_{1}+18x_{2}<33$$

implies that the probability of belonging to pathological group is greater than the probability of belonging to the normal group.

Every positive integer multiple of these numbers will have the same property. For example: The triple (24, 36, 66) also works.

To calculate this index it is necessary to: (a) multiply alpha/theta of mean potential of C3 electrode by 18; (b) multiply alpha/theta of mean potential of O1 electrode by 12; and (c) sum the two values. A result of <33 indicates dementia else no dementia.

Regarding sensitivity, among subjects classified as AD on the test, the result was correct in 76.4% of cases. In terms of specificity among subjects classified as Normal on the test, the result was correct in 84.6% of cases.

The formula yielded 86 correct diagnoses out of 107 cases, or an overall rate of correct diagnosis of 80.4%. These variables explained 80.4% of Pathology, 2.8% higher than the model containing only one variable which yielded 77.6% correct diagnosis.

## Discussion

Although numerous studies in the literature have shown correlation of progressive slowing of EEG waves with AD evolution (Brenner et al., 1988; Watanabe et al., 1993; Anderer et al., 1994; Claus et al., 1998; Babiloni et al., 2004) and reported lower alpha/theta ratios in AD (Cibils, 2002; Reis and Fonseca, 2010), they were based on groups of patients and not on healthy individuals “*per se*” and also failed to quantify a mean numeric value for the ratio in a group of normal individuals.

Our results showed that it is possible to discriminate AD patients from normal individuals using a mathematical analysis tools in combination with EEG. A cut-off index distinguishing between these two samples with 76.4% sensitivity and 84.6% specificity was determined. These findings discriminated among individuals as well as between the AD and control groups, what represents advancement.

In the present study, a cut-off value of 33 for logistic regression of the alpha/theta ratio was determined. Thus, individuals with results ≥33, have a high electroencephalographic probability of being normal, whereas for values lower than 33 there is a high probability of the subject belonging to the AD group.

Like most current methods of diagnosis, even more in the case of functional method, the correlation was made and is significant compared to the clinical findings. The gold standard for AD’s diagnosis is still pathological. Therefore, the method presented in here is not a definitive tool but an instrument as an aid in diagnosis in search of a non-invasive biomarker.

These results numerically corroborate findings in the literature, i.e., that among normal individuals there is a predominance of posterior alpha activity over slow activity (theta), while this predominance diminishes with slowing of the trace and increased theta activity relative to lower alpha activity. The anatomo-physiological substrate strongly supporting these findings is that of the cholinergic theory for AD.

Changes in the cholinergic system were studied in normal aging and in AD (Schliebs and Arendt, 2011) leading to evidence of severe deficits in pre-synaptic cholinergic markers in cerebral cortex of AD patients (Davies and Maloney, 1976). This theory was corroborated by studies in the literature showing correlation between cholinergic hypofunction and cognitive deficits, leading to the cholinergic hypothesis to explain decline in memory among normal elderly and in AD (Mufson et al., 2008).

Patients with AD have a significantly less complex EEG signal than normal individuals of similar age. This finding can be associated with impaired processing in brains with AD, characterized by a loss of modulation and complexity of brain rhythms, reflecting impaired neurotransmission. The majority of studies on EEG in AD have shown marked decline in coherence of the alpha band, which have been associated with increased genetic risk of ApoE, hypothetically related to impaired cholinergic neurotransmission (Lizio et al., 2011).

The increased relative theta potential described is not new in the literature, it is in fact expected (Bennett et al., 2004). From a functional standpoint, the increased relative potential of the theta band is a sign of medial hippocampal activation while the decrease in alpha activity correlates with involvement of the corticothalamo-cortical retroactivation mechanism (Colom, 2006).

These literature findings of thalamic dysfunction in cortical connectivity highlight the need to scrutinize the information which advances in mathematics and computation can derive from electroencephalographic records. It is known that the thalamus, together with the cuneus and precuneus, are the main sources of alpha activity of patients at rest (Cantero et al., 2009). Slowing of the alpha rhythm and increasing of theta activity can occur in a broad array of neurological and psychiatric disorders (Llinás et al., 1999).

Based on these postulations in the literature, we cannot categorically affirm that our findings confirm a definitive marker for discriminating AD patients from normal individuals. Nevertheless, this index of logistic regression for the alpha/theta ratio can be used to indicate a watershed between normality and individuals with pathologies that reflect disturbances in brain function through the non-invasive EEG exam.

## Conclusion

To summarize, logistic regression of the alpha/theta ratio of the spectrum of the mean potential of EEG constitutes a valuable marker for discriminating AD patients from healthy controls, where individuals with an index ≥33 are considered electroencephalographically normal.

There is no established markers for the EEG, and more than having a marker for AD, the first point would have a marker for normal, which would be a great starting point for future increment methodology.

Undoubtedly, our findings regarding the pathology already has its importance by itself, but we believe that the biggest breakthrough refers to the existence of a normality cut-off, statistically robust, which had not yet in quantitative EEG.

Further investigations are needed to confirm these findings and render this EEG analysis tool more robust, hopefully promoting its future use in routine practice.

## Conflict of Interest Statement

The authors declare that the research was conducted in the absence of any commercial or financial relationships that could be construed as a potential conflict of interest.
